# Subanesthetic doses of ketamine to rats and monkeys rapidly increases radioligand binding in brain to phosphodiesterase-4, an indirect marker of cAMP

**DOI:** 10.1038/s41398-026-04039-w

**Published:** 2026-04-21

**Authors:** Paul A. Parcon, Amanda Bardhoshi, Amanda Olsen-Dufour, Raven Cureton, Susovan Jana, Cheryl L. Morse, Ioline D. Henter, Julia Totis, Adrian E. Jenson, Matilah T. Pamie-George, Jeih-San Liow, Sami S. Zoghbi, Shawn Wu, Victor W. Pike, Robert B. Innis

**Affiliations:** Molecular Imaging Branch, National Institute of Mental Health, National Institutes of Health, Bethesda, MD USA

**Keywords:** Biomarkers, Neuroscience, Depression

## Abstract

The *N*-methyl-D-aspartate (NMDA) receptor antagonist ketamine is a robust, rapid-acting antidepressant whose molecular effects have not been fully elucidated. Phosphodiesterase-4 (PDE4), which terminates cyclic adenosine monophosphate (cAMP) activity, may underlie antidepressant response. In particular, previous studies found that whole brain binding of the positron emission tomography (PET) radioligand [^11^C](*R*)-rolipram to PDE4 was decreased in individuals with major depressive disorder; eight weeks of antidepressant treatment rescued this decrease in [^11^C](*R*)-rolipram binding. This study used [^11^C](*R*)-rolipram, which targets all PDE4 subtypes, and [^18^F]PF-06445974, which is preferential for PDE4B over PDE4D, to determine whether ketamine infusion could rapidly increase cAMP activity in rats (at 10 mg/kg) and rhesus macaques (at 0.5 mg/kg). Ketamine increased [^11^C](*R*)-rolipram binding to PDE4 in rats (mean standardized uptake value (SUV) increase=24% ± 14%, range=3%-42%, *p* = 0.004) and in monkeys (mean distribution volume (*V*_T_) increase=14% ± 2%, range=12%-16%, *p* = 0.003). Ketamine also increased [^18^F]PF-06445974 binding in monkeys within one hour of infusion (mean *V*_T_ increase 28% ± 7%, range=16%-37%, p = 0.008). When [^11^C](*S*)-rolipram, which has no specific binding to PDE4, was used to control for the effects of ketamine on blood flow and radioligand delivery in rats, no consistent effects were observed for ketamine. Collectively, the results suggest that ketamine infusion rapidly increases cAMP activity and may be an underlying mechanism for ketamine’s rapid antidepressant effects. These data support a common pathway for cAMP and antidepressant action and suggest that PDE4 inhibition, particularly PDE4B, may be an effective and rapid-acting antidepressant mechanism.

## Introduction

Subanesthetic doses of ketamine, an *N*-methyl-D-aspartate (NMDA) receptor antagonist, have demonstrated remarkable efficacy in rapidly alleviating depressive symptoms in treatment-resistant depression (TRD), often within hours of administration [1, 2]. The growing interest in ketamine as a rapid-acting antidepressant has also prompted extensive exploration of its neurobiological mechanisms [3, 4]. In this context, cyclic adenosine monophosphate (cAMP) has emerged as a critical player among the various signaling pathways implicated in mood regulation.

cAMP is a second messenger involved in numerous physiological processes, including neurotransmitter release, neuronal excitability, and synaptic plasticity [3, 5]. Dysregulation of cAMP signaling has been associated with mood disorders, making it a target of interest in psychiatric research [6], and different classes of antidepressants have been shown to increase cAMP activity [7]. Although ketamine has been shown to enhance synaptic connectivity and promote neuroplasticity [8, 9], it remains unclear whether it directly alters cAMP activity. Nevertheless, recent studies suggest that ketamine and its metabolites may increase cAMP levels [10], potentially leading to downstream effects that support its antidepressant properties.

The phosphodiesterase 4 (PDE4) class of enzymes regulates cAMP via hydrolysis to 5’-AMP, making it a prime target for drug development [11]. Several PDE4 inhibitors have been tested as antidepressants, particularly the pan-PDE4 subtype inhibitor rolipram [12, 13], though its efficacy was limited by significant side effects (e.g., nausea, vomiting). A previous study from our laboratory found that a radiolabeled version of rolipram, [^11^C](*R*)-rolipram, was a sensitive probe of cAMP activity due to negative feedback mechanisms [14, 15]; specifically, cAMP led to increased protein kinase A (PKA) phosphorylation that, in turn, led to increased radioligand affinity. Building on this work, another study from our laboratory found that unmedicated participants with major depressive disorder (MDD) currently experiencing a major depressive episode had significantly reduced [^11^C](*R*)-rolipram uptake compared to healthy volunteers [16]. In addition, eight weeks of treatment with selective serotonin reuptake inhibitors (SSRIs) re-normalized [^11^C](*R*)-rolipram uptake, although this notably did not correlate with response rates on clinical rating scales [17].

Subtype specificity for PDE4 may contribute to this lack of correlation between normalized [^11^C](*R*)-rolipram uptake and clinical rating scales. As a pan-PDE4 radioligand, [^11^C](*R*)-rolipram would be expected to bind to all four subtypes of PDE4 (PDE4A, PDE4B, PDE4C, and PDE4D). These subtypes differ in their localization, expression, and regulation but share the enzymatic ability to hydrolyze and thereby terminate cAMP. In particular, PDE4B inhibition has been shown to have both anti-inflammatory and antidepressant effects in animal models, suggesting that it may be the primary subtype associated with depression. Our laboratory recently developed [^18^F]PF-06445974, a radioligand preferential for PDE4B over PDE4D (and to lesser extent, over PDE4A), and found that it has appropriate characteristics for both human and animal imaging [18].

As noted above, unmedicated individuals with MDD experiencing a major depressive episode had decreased uptake of [^11^C](*R*)-rolipram, a surrogate marker of cAMP activity, of around 20% compared to healthy volunteers; this effect was reversed after eight weeks of SSRI treatment [16, 17]. The present study sought to use [^11^C](*R*)-rolipram and the PDE4B-preferential radioligand [^18^F]PF-06445974 to determine how infusions of subanesthetic-dose ketamine affect cAMP activity and rolipram binding. Towards this end, PET neuroimaging was conducted with two radioligands—[^11^C](*R*)-rolipram and [^18^F]PF-06445974—in rats and monkeys. The results will help elucidate whether ketamine’s rapid antidepressant effects may include an increase in cAMP activity.

## Materials and methods

### Radiosynthesis

[^11^C](*R*)-rolipram was synthesized as previously described [19] and had molar activity (*A*_M_) at time of injection of 3.14 ± 1.33 Ci/µmol and a radiochemical purity greater than 99%. [^11^C](*S*)-Rolipram was synthesized as previously described [19] and had molar activity (*A*_M_) at time of injection of 4.44 ± 1.29 Ci/µmol and a radiochemical purity greater than 99%. [^18^F]PF-06445974 was synthesized as previously described [18] and had molar activity (*A*_M_) at time of injection of 2.34 ± 0.92 Ci/µmol and a radiochemical purity of >99%. All three radioligands were formulated for intravenous injection in sterile saline containing 10% v/v ethanol.

### Animals

The experiments were conducted in accordance with the Guide for Care and Use of Laboratory Animals [20] and were approved by the Animal Care and Use Committee of the National Institute of Mental Health (NIMH) under protocol numbers MIB-02 for nonhuman primates and MIB-03 for rats. All animals were included in the analysis, and no pre-determined sample size was calculated. No randomization was included in any study, and the study was not blinded. Adult male Sprague-Dawley rats about 2 months old were anesthetized with isoflurane, and an intravenous catheter was placed in the penile vein, which was used for both radioligand infusion and ketamine infusion. At baseline, six rats were imaged with [^11^C](*R*)-rolipram, and five rats were imaged with [^11^C](*S*)-rolipram; the latter has no specific binding to PDE4 and was used as a negative control. To assess the effects of ketamine on cAMP activity, the same rats were subsequently infused with subanesthetic ketamine (10 mg/kg for one hour) then imaged with the same radioligand within one hour of the end of the ketamine infusion. Following injection of the radioligand, rats were imaged as previously described [21] in the Mediso LFER 150 PET/CT scanner for 90 min. Rat imaging was corrected for body weight and injected dose to express radioactivity concentration as a standardized uptake value (SUV). Data are reported as area-under-the-curve for the last hour (AUC_30-90_) in units of (SUV•min).

Adult monkeys were initially imaged at baseline with [^11^C](*R*)-rolipram (one male, two female) or [^18^F]PF-06445974 (two male, two female), then imaged again after 0.5 mg/kg IV ketamine infusion over 40 min for comparison. Three of the same animals were used for both experiments, and one additional monkey was used in the [^18^F]PF-06445974 experiments. Following injection of the radioligand, monkeys were imaged as previously described [18] in a Mediso LFER 150 PET/CT scanner for two hours. The post-ketamine scan occurred within one hour of the end of the 40-minute ketamine infusion. Serial arterial blood samples were collected via an indwelling catheter in the femoral artery to measure radioactivity in the blood for both pre- and post-ketamine PET scans. The concentration over time of the parent radioligand separated from radiometabolites was used as the input function to regional brain uptake using a two-tissue compartment model. These data were used to calculate total volume of distribution (*V*_T_) reported in mL/cm^3^. Representative parametric images of *V*_T_ were calculated using Logan graphical analysis in PXMOD.

### Administration of ketamine

Racemic ketamine was diluted from 100 mg/mL stock solution to a working solution of 10 mg/mL in sterile normal saline immediately prior to the ketamine infusion. Ketamine was infused intravenously to rats as a single dose of 10 mg/kg for 40 min and to monkeys at a single dose of 0.5 mg/kg for 40 min IV, which is equivalent to human antidepressant dosing. Ketamine did not affect measured vital signs in monkeys, including blood pressure, mean arterial pressure, pulse, or blood oxygen saturation.

### Statistics

The primary endpoint for all studies was change in whole brain uptake of radioligand using within-subjects paired two-tailed *t*-tests with α = 0.05. *V*_T_ values are presented as mean ± SD. The Shapiro-Wilk test did not show significant departure from normality for all groups. Percent difference calculations were performed for specific brain areas in rhesus monkeys, but this study was not powered to control for multiple hypothesis testing for each brain area.

## Results

### Ketamine infusion increased [^11^C](R)-rolipram uptake in rat brain

**Fig. 1:**
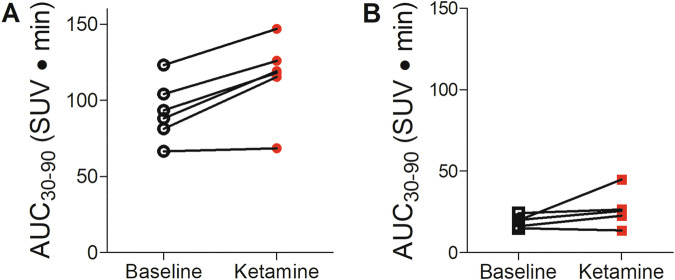
Ketamine infusion increases [11C](R)-rolipram uptake in rat brain. **A** [^11^C](*R*)-rolipram binding levels at baseline (black, open circles) and one hour after 10 mg/kg IV ketamine infusion in rats (n = 6) (red, closed circles) measured as area under the curve from 30–90 min (AUC_30-90_) (standardized uptake value (SUV)*min) in whole brain. **B** [^11^C](*S*)-rolipram, which has no specific binding to PDE4, was used as a control for changes in blood flow at baseline (black, open squares) and one hour after 10 mg/kg IV ketamine infusion in rats (n = 5) (red, closed squares), measured as AUC_30-90_ (SUV*min) in whole brain. Rats treated with [^11^C](*R*)-rolipram demonstrated a consistent increase of about 23 SUV*min on average (95% confidence interval 11.06 to 34.62; p = 0.0042, paired *t*-test).

To determine the effects of ketamine infusion on cAMP activity, six rats were imaged with [^11^C](*R*)-rolipram at baseline and after ketamine infusion (10 mg/kg over 40 min). For each animal measured, the post-ketamine scan showed increased uptake of [^11^C](*R*)-rolipram compared to baseline (115.72 to 92.88 SUV*min, p = 0.0042, two-tailed paired *t*-test, Fig. 1A). Because [^11^C](*S*)-rolipram does not bind with high affinity to PDE4, an additional five rats were also scanned at baseline and post-ketamine with [^11^C](*S*)-rolipram, which showed much less uptake as well as no significant increase in uptake post-ketamine (26.72 to 19.13 SUV*min, *p* = 0.19, two-tailed paired *t*-test, Fig. 1B). These results are consistent with a ketamine-induced increase of cAMP activity in rats.

### Ketamine infusion increased [^11^C](R)-rolipram uptake in monkey brain

**Fig. 2:**
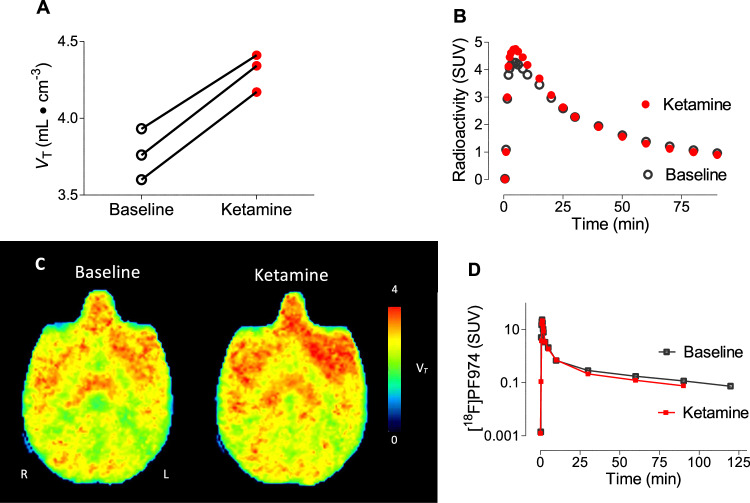
Ketamine infusion increased [^11^C](R)rolipram uptake in monkey brain. **A** [^11^C](*R*)-rolipram binding levels at baseline (black, open circles) and one hour after 0.5 mg/kg IV ketamine infusion in monkeys (n = 3) (red, closed circles) measured as total distribution volume (*V*_T_), calculated with two-tissue compartmental modeling. All three monkeys demonstrated a consistent increase of 0.54 (95% confidence interval 0.41 to 0.68) on average (p = 0.0034, paired *t*-test). **B** Representative time activity curve for whole brain before (black, open circles) and one hour after ketamine infusion in monkeys (red, closed circles). **C** Representative parametric images before (left) and after ketamine infusion (right) in monkey brain. **D** Representative time activity curve for plasma parent before (black, open squares) and one hour after ketamine infusion in monkey (red, closed squares).

Given possible differences between rodents and primates, further experiments were performed on three rhesus macaques. Rhesus macaques were imaged with [^11^C](*R*)-rolipram both at baseline and within one hour of ketamine infusion (0.5 mg/kg over 40 min, equivalent to a human antidepressant dose). The primary endpoint was whole brain uptake of [^11^C](*R*)-rolipram, measured as *V*_T_, determined by two-tissue compartmental modeling with arterial input function. In all three monkeys, [^11^C](*R*)-rolipram uptake was increased in the whole brain post-ketamine infusion (3.76 ± 0.17 to 4.3 ± 0.12 mL/cm^3^, *p* = 0.0034, two-tailed paired *t*-test, Fig. 2A); representative time-activity curves for the brain are shown in Fig. 2B, representative parametric images are shown in Fig. 2C, and representative arterial input function is shown in Fig. 2D. Percentage differences were consistent across animals, with an average of 14% ± 2%. When [^11^C](*R*)-rolipram was separately measured in 11 parcellated brain regions based on the magnetic resonance imaging (MRI) brain atlas (see Table 1), the percentage differences between brain areas was remarkably consistent, with values between 12% and 18%; the highest values were observed in the striatum and the lowest values were observed in the hippocampus and thalamus (12%). Thus, ketamine’s effects on [^11^C](*R*)-rolipram appeared to be consistent and brain-wide.

**Table 1:** Ketamine increased the *V*_T_ of [^11^C](*R*)-rolipram in monkey brain (n = 3).

Region	Baseline (mL • cm^−3^)	Ketamine (mL • cm^−3^)	%Δ
Whole Brain	3.76	4.31	14%
Frontal Cortex	4.04	4.57	13%
Anterior Cingulate	4.06	4.74	17%
Striatum	3.99	4.72	18%
Insula	4.14	4.73	14%
Temporal Cortex	3.44	3.96	15%
Amygdala	3.68	4.25	16%
Hippocampus	3.80	4.27	12%
Thalamus	4.09	4.60	12%
Parietal Cortex	4.05	4.59	13%
Occipital Cortex	3.73	4.26	14%
Cerebellum	2.89	3.39	17%

*V*_T_: distribution volume.

### Ketamine infusion increased [^18^F]PF-06445974 uptake in monkey brain

**Fig. 3:**
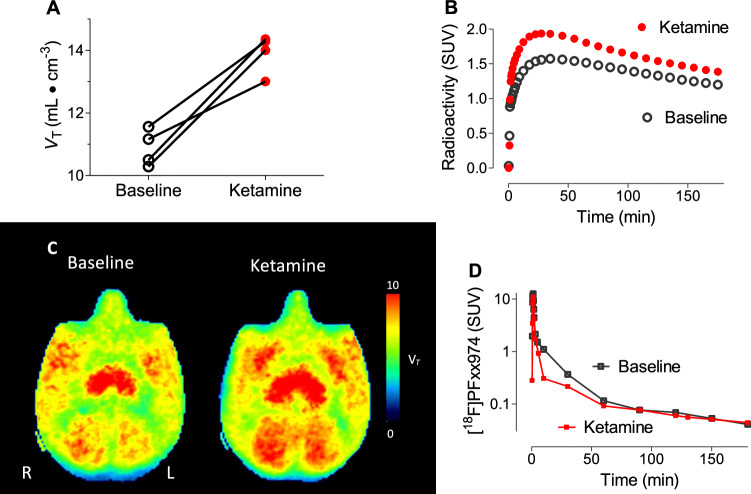
Ketamine infusion increased [^18^F]PF-06445974 uptake in monkey brain. **A** [^18^F]PF-06445974 binding levels at baseline (black, open circles) and one hour after 0.5 mg/kg IV ketamine infusion in monkeys (n = 4) (red, closed circles) measured as total distribution volume (*V*_T_), calculated with two-tissue compartmental modeling. All four monkeys demonstrated a consistent increase of 3.031 (95% confidence interval 1.5 to 4.5) on average (p = 0.0076, paired *t*-test). **B** Representative time activity curve for whole brain before (black, open circles) and one hour after ketamine infusion in monkeys (red, closed circles). **C** Representative parametric images before (left) and after ketamine infusion (right) in monkey brain. **D** Representative time activity curve for plasma parent before (black, open squares) and one hour after ketamine infusion in monkeys (red, closed squares).

Because PDE4B is hypothesized to be the primary antidepressant subtype of PDE4, the effects of ketamine on the PDE4B-preferring radioligand [^18^F]PF-06445974 were assessed in four monkeys; three of these were the same animals who received [^11^C](*R*)-rolipram. The monkeys were imaged both at baseline and within one hour of ketamine infusion. As with [^11^C](*R*)-rolipram, an increase in whole brain *V*_T_ was observed after ketamine infusion in every animal (from 10.88 ± 0.59 to 13.91 ± 0.62, p = 0.0076, two-tailed paired *t*-test, Fig. 3A); representative time-activity curves for brain are shown in Fig. 3B, representative parametric images are shown in Fig. 3C, and arterial input function is shown in Fig. 3D. Whole brain percentage differences showed greater variability, with an average of 28.25% ± 10.08%. Significant variability was also evident after parcellation, with percentage differences in different brain areas ranging from 14% to 74% (Table 2).

**Table 2:** Ketamine increased the *V*_T_ of [^18^F]PF-06445974 in monkey brain (n = 4).

Region	Baseline (mL • cm^−3^)	Ketamine (mL • cm^−3^)	%Δ
Whole Brain	10.88	13.91	28%
Frontal Cortex	10.64	16.16	52%
Anterior Cingulate	11.1	12.83	16%
Striatum	11.53	20.02	74%
Insula	11.58	14.99	29%
Temporal Cortex	9.335	10.84	16%
Amygdala	9.211	11.19	21%
Hippocampus	11.58	17.26	49%
Thalamus	12.92	14.69	14%
Parietal Cortex	11.02	13.13	19%
Occipital Cortex	10.62	13.18	24%
Cerebellum	9.66	12.37	28%

*V*_T_: distribution volume.

## Discussion

This PET study used [^11^C](*R*)-rolipram and the PDE4B-preferential radioligand [^18^F]PF-06445974 to determine how subanesthetic-dose ketamine infusions affect cAMP activity in rodents and monkeys. In both rats and monkeys, subanesthetic-dose ketamine significantly increased binding of both [^11^C](*R*)-rolipram and [^18^F]PF-06445974 within one hour of infusion. The rapid effects suggest that the increased binding was caused by post-translational modification of the protein rather than new synthesis. Specifically, it appears plausible that ketamine increased cAMP activity, which activated PKA and phosphorylated PDE4, which increased its affinity to bind these radioligands.

As noted previously, rolipram is a reversible PDE4 inhibitor, and [^11^C](*R*)-rolipram binding is positively correlated with cAMP activity. A previous series of rat studies confirmed a negative feedback system mediated by PKA [14, 15]. Within this system, cAMP stimulates PKA, which phosphorylates PDE4, which increases enzyme activity about 10-fold and increases the affinity of rolipram for PDE4 by about 10-fold. Therefore, the in vivo binding of [^11^C](*R*)-rolipram largely reflects the activated (phosphorylated) form of PDE4, which is mediated by cAMP and PKA.

Building on this work, a study from our laboratory used [^11^C](*R*)-rolipram to measure PDE4 binding in six individuals with McCune-Albright Syndrome (MAS); nine healthy volunteers were also scanned [22]. MAS is a mosaic disease arising from gain-of-function mutations in the *GNAS* gene, which encodes the cAMP pathway-associated G-protein, Gsα. Increased cAMP levels have been found in vitro in both animal models of fibrous dysplasia [23] and in cultured cells from individuals with MAS [24]. Our study, which compared whole-body scan uptake between areas of fibrous dysplasia and unaffected bones, found that [^11^C](*R*)-rolipram binding correlated with known locations of fibrous dysplasia in the periphery of individuals with MAS; no uptake was observed in the bones of healthy volunteers. In peripheral organs and in the brain, no difference in [^11^C](*R*)-rolipram uptake was observed between individuals with MAS and healthy volunteers. Collectively, these results provided the first evidence of increased cAMP activity in areas of fibrous dysplasia in humans in vivo, as well as further confirmation that [^11^C](*R*)-rolipram uptake can indirectly measure cAMP activity.

Previous studies from our laboratory also found that [^11^C](*R*)-rolipram binding was globally decreased in unmedicated individuals with MDD experiencing a major depressive episode compared to healthy volunteers [16]. Furthermore, two months of treatment with an SSRI in individuals with MDD increased (normalized) [^11^C](*R*)-rolipram binding compared to pretreatment values [17]. These results suggested that chronic treatment with antidepressants increased the cAMP signaling cascade, including PDE4. This observation lends credence to the hypothesis that low cAMP activity may correlate with depressive symptoms, and that correcting cAMP deficits may be a therapeutic target in mood disorders. Importantly, ketamine exerts much more rapid antidepressant effects than SSRIs, consistent with the results presented here.

The mechanism(s) of ketamine’s antidepressant effects remain unclear, but at least two laboratories have suggested that they may include increased cAMP activity. Using C6 glioma cells, one study found that both ketamine and its metabolite (2*R*,6*R*)-hydroxynorketamine ((2*R*,6*R*)-HNK) redistributed Gαs in the membrane to increase cAMP production [10]. This effect persisted after knocking out the NMDA receptor, suggesting that the pathway is NMDA receptor-independent. The same investigators had previously found that several classes of antidepressants similarly affected C6 glioma cells, suggesting that increased cAMP activity is a common pathway for both ketamine and conventional antidepressants [25]. In that study, the administration of SSRIs to MDD participants and the administration of racemic ketamine in animal models both increased cAMP activity as measured by radioligand binding; these increases occurred within the time frames expected for their antidepressant action, consistent with the notion of a common pathway.

Unlike racemic ketamine, (2*R*,6*R*)-HNK appears to exert its antidepressant effects without significantly affecting NMDA receptors [3]. In this vein, Riggs and colleagues examined presynaptic potentiation in rat hippocampal slices as a model for ketamine’s prolonged antidepressant effects [26]. Although ketamine was inactive in their in vitro system, (2*R*,6*R*)-HNK increased synaptic efficacy, suggesting that (2*R*,6*R*)-HNK—rather than the parent drug ketamine—mediated this effect. Of special note for the current study, Brown and colleagues further found that presynaptic potentiation was blocked by inhibitors of adenylyl cyclase and PKA [27]. This suggests that increased cAMP activity is needed for presynaptic potentiation of (2*R*,6*R*)-HNK. Our use of PDE4 radioligand binding as an indirect measure of cAMP activity is consistent with these results. That is, intravenously-administered ketamine, which breaks down into (2*R*,6*R*)-HNK, increased the binding of two PDE4 radioligands thought to correlate with cAMP activity.

It is notable that ketamine’s effects on [^18^F]PF-06445974 were greater than its effects on [^11^C](*R*)-rolipram. Interestingly, Zhang and colleagues hypothesized that PDE4B inhibition, in particular, has antidepressant effects [28]. A small study that investigated the antidepressant effects of [^11^C](*R*)-rolipram found no correlation with clinical response [17], but the ability of [^11^C](*R*)-rolipram to bind to all four PDE4 subtypes may have led to a “washing-out” effect. In this context, clinical rating scales may instead correlate with PDE4B-specific changes. Towards that end, [^18^F]PF-06445974, which is preferential for PDE4B over PDE4D, has been tested in human participants [18]. Because ketamine appears to affect [^18^F]PF-06445974 binding, it is expected that future studies in individuals with MDD may demonstrate greater effects than those seen with [^11^C](*R*)-rolipram.

This study also found that ketamine increased [^11^C](*R*)-rolipram and [^18^F]PF-06445974 binding throughout the brain. Such an effect is not unexpected, as NMDA transmission and PDE4B distribution are widespread, although with some local variability. While this widespread effect does not necessarily reflect specific depression-related circuits, this does not negate the possibility that ketamine’s mechanism of action includes increasing cAMP activity. It is possible, for example, that while ketamine’s effects on cAMP are widespread, only some regions and circuits may mediate any putative antidepressant effects due to their particular vulnerability to changes in cAMP, or due to the translation of cAMP changes to a behavioral or psychological phenotype. It is plausible that circuit-related as well as circuit-independent phenotypes contribute to syndromic depression, and these may not be fully measured by a single imaging scan. For example, in the previous study of depressed participants [17], the average [^11^C](*R*)-rolipram uptake may have been lower, but it may be that individuals with lower [^11^C](*R*)-rolipram uptake may have had other compensatory resilience factors that resulted in them not presenting as clinically depressed; conversely, a depressed individual with higher [^11^C](*R*)-rolipram uptake may have had other vulnerabilities.

Despite these intriguing results, a number of limitations warrant attention. First, the animals were healthy, wild-type animals rather than animal models of depression; it is possible that the observed effects would be greater in animal models of depression. Second, because these animals received only a single ketamine infusion, the results may not capture longer-lasting effects or effects associated with repeated dosing. Third, the sample sizes were small (only 11 rats and four monkeys), which reflects the fact that PET is an expensive and scarce resource. Fourth, only male rodents were used; however, this limitation is offset by our having used both male and female monkeys, an animal model with greater translational proximity to humans. Finally, this study was not appropriately powered to control for multiple hypothesis testing of changes in each individual brain area, as kinetic modeling of smaller brain areas comes with greater variability. Nevertheless, radioligand uptake generally followed the trends previously published by Perez-Torres and colleagues [29], demonstrating PDE4B enrichment (for example, in the striatum).

Despite these limitations, it should be noted that this work was designed as a proof-of-principle study in animals prior to a potential study using the PDE4B-preferring radioligand [^18^F]PF-06445974 before and after ketamine administration in individuals with MDD. In the opinion of the authors, these animal results—in combination with numerous other studies of PDE4 and cAMP—adequately justify a proposed study in individuals with MDD to evaluate whether PDE4B inhibitors could potentially be used to treat depression. In particular, the results would help elucidate whether a subset of individuals with depression have low cAMP activity and, if so, whether treatment with a PDE4B inhibitor could increase cAMP activity and improve depressive symptoms. Importantly, the nausea and emesis previously found in antidepressant trials of the pan-PDE4 inhibitor rolipram are thought to be mediated by PDE4D inhibition. Thus, a PDE4B selective inhibitor would not be expected to cause nausea and vomiting. Indeed, two recent reports of a PDE4B inhibitor used to successfully treat inflammation in idiopathic pulmonary fibrosis found that nausea and emesis were minimal, with similar prevalence to the placebo group [30, 31].

### Conclusions and future directions

Ketamine rapidly increased cAMP activity in rodent and monkey brain, as indirectly assessed by increased radioligand binding to PDE4 measured via the pan-PDE4 radioligand [^11^C](*R*)-rolipram and the PDE4B preferential radioligand [^18^F]PF-06445974. Collectively, the results suggest that ketamine’s rapid antidepressant effects may be mediated in part by increased cAMP activity. Future studies in individuals experiencing a major depressive episode are planned to investigate whether PDE4B radioligand binding is decreased in these individuals and whether ketamine treatment would rapidly increase PDE4B radioligand binding.

## Data Availability

The data that support the findings of this study are available on OpenNeuro (https://openneuro.org/datasets/ds007653).
